# Asymptotic Synchronization of Memristive Cohen-Grossberg Neural Networks with Time-Varying Delays via Event-Triggered Control Scheme

**DOI:** 10.3390/mi13050726

**Published:** 2022-04-30

**Authors:** Wei Yao, Fei Yu, Jin Zhang, Ling Zhou

**Affiliations:** 1 School of Computer and Communication Engineering, Changsha University of Science & Technology, Changsha 410114, China; yaowei520026@sina.com (W.Y.); yufeiyfyf@csust.edu.cn (F.Y.); 2 Key Laboratory of Industrial Control Technology, Zhejiang University, Hangzhou 310058, China; 3 School of Intelligent Manufacturing, Hunan University of Science and Engineering, Yongzhou 425199, China

**Keywords:** asymptotic synchronization, memristive Cohen-Grossberg neural network, event-triggered control, time-varying delays

## Abstract

This paper investigates the asymptotic synchronization of memristive Cohen-Grossberg neural networks (MCGNNs) with time-varying delays under event-triggered control (ETC). First, based on the designed feedback controller, some ETC conditions are provided. It is demonstrated that ETC can significantly reduce the update times of the controller and decrease the computing cost. Next, some sufficient conditions are derived to ensure the asymptotic synchronization of MCGNNs with time-varying delays under the ETC method. Finally, a numerical example is provided to verify the correctness and effectiveness of the obtained results.

## 1. Introduction

In the past few decades, complex systems including neural networks (NNs) have been extensively studied due to their wide applications [1,2,3,4,5]. Based on the excellent characteristics of memristor [6,7,8], a variety of chaotic circuits and systems based on memristor are proposed. Memristive neural network (MNN), which simulates synaptic connection with memristor, has attracted much attention owing to its application in logic operation and image processing [9,10,11,12,13,14,15,16].

The Cohen-Grossberg neural network is a generalized neural network model, which can take famous neural networks and systems such as the Hopfield neural network and Lotka–Volterra system as its special cases [17,18,19]. In recent years, memristive Cohen-Grossberg neural network (MCGNN) and its dynamical characteristics have attracted increasing attention [20,21,22,23]. In [21], there exist exponentially stable equilibrium points in n-dimensional MCGNNs with piecewise linear activation functions via the fixed point theorem. Global exponential stability of delayed and perturbed MCGNNs was investigated in [22]. Paper [23] studied multistability of MCGNNs with mixed delays and acquired multiple almost periodic solutions. Synchronization as one of the most important dynamical characteristics has been researched extensively and many papers on synchronization of MCGNNs have been published [24,25,26,27,28]. In [24], Yang et al. investigated global exponential synchronization of MCGNNs with time-varying discrete delays and unbounded distributed delays. Wei et al. studied fixed-time synchronization of MCGNNs with impulsive effects [25]. Ren et al. investigated finite-time synchronization and quasi fixed-time synchronization of MCGNNs with reaction-diffusion term in [27,28], respectively. Achieving the synchronization of MCGNNs means that synchronization of multiple classes of neural networks can be acquired, thus being highly important for achieving the synchronization of MCGNNs.

At present, network control schemes include the state-feedback control method and nonlinear control method, which have been widely used in many fields due to their advantages of reliability and high efficiency [29,30,31,32]. However, these network control schemes for MNN synchronization are based on continuous-time feedback controllers [29,30,31,32], thus they require high computing power. As an important sampling control scheme, event-triggered control (ETC) [33,34,35,36,37,38,39,40,41] can effectively reduce computing costs and communication resources on the basis of ensuring system performance by reducing controller update times. Therefore, ETC schemes for MNN synchronization have been extensively studied [33,34,35,36,37,38,39,40,41]. In [36], the stability of MNNs with communication delays was addressed via the event-triggered sampling control method. Using event-triggered impulsive control, quasi-synchronization of delayed MNNs was investigated [37]. In [38], static or dynamic ETC methods were designed to achieve synchronization of delayed MNNs. Some different static or dynamic ETC methods were provided to further realize the synchronization of inertial MNNs [39]. In [40], exponential mean-square stability of delayed discrete-time stochastic MNNs was achieved by an event-triggered H*∞* state estimation. However, these ETC schemes were considered in the traditional MNN system [33,34,35,36,37,38,39,40,41]. In other words, the existing ETC methods cannot be directly used in the synchronization of MCGNNs which increases the difficulty of control and analysis on account of the amplification function of MCGNNs. To the best our knowledge, there is scarce literature regarding synchronization of MCGNN via ETC scheme.

Inspired by the discussion above, this paper investigates the synchronization of MCGNNs with time-varying delays via ETC scheme for the first time. We summarize the main contributions as follows.

(1) This paper designs a state-feedback controller, and some ETC conditions were provided based on the state-feedback controller.
(2) Some sufficient conditions are presented to guarantee asymptotic synchronization of MCGNNs with time-varying delays under ETC condition.
(3) Furthermore, the MCGNNs under ETC schemes can effectively reduce the update times of controllers and decrease computing cost.

The rest of the paper is organized as follows. In Section 2, MCGNNs with time-varying delays are introduced. Some sufficient conditions are obtained to achieve asymptotic synchronization of MCGNNs in Section 3. Section 4 presents a numerical simulation to verify the effectiveness of the obtained results. Finally, conclusions are provided in Section 5.

## 2. Preliminaries

Notations: For a given vector $a={\left(a_{1},a_{2},\ldots ,a_{l}\right)}^{T}$, ${∥a∥}_{1}=\sum_{m=1}^{l}\left|a_{m}\right|$. For a given matrix $x={\left[x_{mn}\right]}_{l\times l}$, ${∥x∥}_{1}=\max_{1\leq n\leq l}\sum_{m=1}^{l}\left|x_{mn}\right|$.

We consider memristive Cohen-Grossberg neural networks (MCGNNs) with time-varying delays as follows.

(1) $$\begin{array}{c} {\dot{r}}_{m}\left(t\right)=a_{m}\left(r_{m}\left(t\right)\right)\left\{-b_{m}r_{m}\left(t\right)+\sum_{n=1}^{l}c_{mn}\left(r_{m}\left(t\right)\right)f_{n}\left(r_{n}\left(t\right)\right))\right.\\ \left.+\sum_{n=1}^{l}d_{mn}\left(r_{m}\left(t\right)\right)f_{n}\left(r_{n}\left(t-\tau_{mn}\left(t\right)\right)\right)+I_{m}\right\},\;m=1,2,\ldots ,l, \end{array}$$

where $r_{m}\left(t\right)$ represents the state of the *m*th neuron; $a_{m}\left(r_{m}\left(t\right)\right)$ and $b_{m}>0$ are the amplification function and behaved function, respectively; $f_{n}\left(\cdot \right)$ is the activation function; $\tau_{mn}\left(t\right)$ denotes time-varying delay and satisfies $0\leq \tau_{mn}\left(t\right)\leq \tau$, where τ is a positive constant; $I_{m}$ is external input; $c_{mn}\left(r_{m}\left(t\right)\right)$ and $d_{mn}\left(r_{m}\left(t\right)\right)$ denote memristive connection weights satisfying the following conditions

(2) $$c_{mn}\left(r_{m}\left(t\right)\right)=\left\{\begin{array}{c} c_{mn}^{\left(1\right)},\;\left|r_{m}\left(t\right)\right|\leq \chi_{m},\\ c_{mn}^{\left(2\right)},\;\left|r_{m}\left(t\right)\right|>\chi_{m}, \end{array}\right.$$

(3) $$d_{mn}\left(r_{m}\left(t\right)\right)=\left\{\begin{array}{c} d_{mn}^{\left(1\right)},\;\left|r_{m}\left(t\right)\right|\leq \chi_{m},\\ d_{mn}^{\left(2\right)},\;\left|r_{m}\left(t\right)\right|>\chi_{m}, \end{array}\right.$$

where $c_{mn}^{\left(1\right)}$, $c_{mn}^{\left(2\right)}$, $d_{mn}^{\left(1\right)}$ and $d_{mn}^{\left(2\right)}$ are constants, $\chi_{m}>0$ is the switching jump.

Set ${\hat{c}}_{mn}=\max\{|c_{mn}^{\left(1\right)}\left|,\right|c_{mn}^{\left(2\right)}\left|\right\}$, ${\bar{c}}_{mn}=\max\left\{c_{mn}^{\left(1\right)},c_{mn}^{\left(2\right)}\right\}$, ${\tilde{c}}_{mn}=\min\left\{c_{mn}^{\left(1\right)},c_{mn}^{\left(2\right)}\right\}$, ${\hat{d}}_{mn}=\max\{|d_{mn}^{\left(1\right)}\left|,\right|d_{mn}^{\left(2\right)}\left|\right\}$, ${\bar{d}}_{mn}=\max\left\{d_{mn}^{\left(1\right)},d_{mn}^{\left(2\right)}\right\}$, ${\tilde{d}}_{mn}=\min\left\{d_{mn}^{\left(1\right)},d_{mn}^{\left(2\right)}\right\}$, $\hat{C}={\left[{\hat{c}}_{mn}\right]}_{l\times l}$ and $\hat{D}={\left[{\hat{d}}_{mn}\right]}_{l\times l}$.

The following assumptions will be used in this paper.

**Assumption** **1.** *Amplification function $a_{m}\left(x\right)$ is continuous and bounded, namely, there exist two positive constants $a_{m}^{\left(1\right)}$ and $a_{m}^{\left(2\right)}$, such that $0<a_{m}^{\left(1\right)}\leq a_{m}\left(x\right)\leq a_{m}^{\left(2\right)}$ for ∀x∈ℜ.*

**Assumption** **2.** *Time-varying delay $\tau_{mn}\left(t\right)$ satisfies*

(4) $${\dot{\tau }}_{mn}\left(t\right)\leq \theta <1.$$

*where θ is a positive constant.*

**Assumption** **3.** *Activation function $f_{n}\left(\cdot \right)$ is bounded and satisfies Lipschitz condition, which means there exist constants $M_{n}$, $L_{n}$ such that $\left|f_{n}\left(s_{1}\right)\right|\leq M_{n}$ for $\forall s_{1}\in ℜ$, and $\left|f_{n}\left(s_{2}\right)-f_{n}\left(s_{3}\right)\right|\leq L_{n}\left|s_{2}-s_{3}\right|$ for any $s_{2}$, $s_{3}\in ℜ$.*

From Assumption 1, there exists the antiderivative of $\frac{1}{a_{m}\left(r_{m}\right)}$. Choose such an antiderivative $h_{m}\left(r_{m}\right)$ which satisfies $h_{m}\left(0\right)=0$. Then $\frac{d}{dr_{m}}h_{m}\left(r_{m}\right)=\frac{1}{a_{m}\left(r_{m}\right)}$. Using the derivative theorem for inverse function, the inverse function $h_{m}^{-1}\left(r_{m}\right)$ of $h_{m}\left(r_{m}\right)$ is differentiable and $\frac{d}{dx_{m}}h_{m}^{-1}\left(x_{m}\right)=a_{m}\left(r_{m}\right)$, where $x_{m}=h_{m}\left(r_{m}\right)$. Set $p_{m}\left(t\right)=h_{m}\left(r_{m}\left(t\right)\right)$, then we can get ${\dot{p}}_{m}\left(t\right)=\frac{{\dot{r}}_{m}\left(t\right)}{a_{m}\left(r_{m}\left(t\right)\right)}$, where $r_{m}\left(t\right)=h_{m}^{-1}\left(p_{m}\left(t\right)\right)$. Substituting these equalities into system (1), we can obtain the MCGNN with time-varying delays as follows.

(5) $$\begin{array}{c} {\dot{p}}_{m}\left(t\right)=-b_{m}h_{m}^{-1}\left(p_{m}\left(t\right)\right)+\sum_{n=1}^{l}c_{mn}\left(h_{m}^{-1}\left(p_{m}\left(t\right)\right)\right)\\ \times f_{n}\left(h_{n}^{-1}\left(p_{n}\left(t\right)\right)\right)+\sum_{n=1}^{l}d_{mn}\left(h_{m}^{-1}\left(p_{m}\left(t\right)\right)\right)\\ \times f_{n}\left(h_{n}^{-1}\left(p_{n}\left(t-\tau_{mn}\left(t\right)\right)\right)\right)+I_{m},\;m=1,2,\ldots ,l. \end{array}$$

For a given set γ⊂ℜ, co[γ] represents the closure of the convex hull for set γ. According to the theory of differential inclusion [42], it can be gained from (5) that

(6) $$\begin{array}{c} {\dot{p}}_{m}\left(t\right)\in -b_{m}h_{m}^{-1}\left(p_{m}\left(t\right)\right)+\sum_{n=1}^{l}co\left[{\tilde{c}}_{mn},{\bar{c}}_{mn}\right]f_{n}\left(h_{n}^{-1}\left(p_{n}\left(t\right)\right)\right)\\ +\sum_{n=1}^{l}co\left[{\tilde{d}}_{mn},{\bar{d}}_{mn}\right]f_{n}\left(h_{n}^{-1}\left(p_{n}\left(t-\tau_{mn}\left(t\right)\right)\right)\right)+I_{m},\;m=1,2,\ldots ,l, \end{array}$$

or equivalently, by the measurable selection theorem in [42], there exist measurable functions $c_{mn}^{*}\left(t\right)\in co\left[{\tilde{c}}_{mn},{\bar{c}}_{mn}\right]$, $d_{mn}^{*}\left(t\right)\in co\left[{\tilde{d}}_{mn},{\bar{d}}_{mn}\right]$, such that

(7) $$\begin{array}{c} {\dot{p}}_{m}\left(t\right)=-b_{m}h_{m}^{-1}\left(p_{m}\left(t\right)\right)+\sum_{n=1}^{l}c_{mn}^{*}\left(t\right)f_{n}\left(h_{n}^{-1}\left(p_{n}\left(t\right)\right)\right)\\ +\sum_{n=1}^{l}d_{mn}^{*}\left(t\right)f_{n}\left(h_{n}^{-1}\left(p_{n}\left(t-\tau_{mn}\left(t\right)\right)\right)\right)+I_{m},\;m=1,2,\ldots ,l, \end{array}$$

Let system (1) as the drive MCGNNs, then the response system can be described as

(8) $$\begin{array}{c} {\dot{z}}_{m}\left(t\right)=a_{m}\left(z_{m}\left(t\right)\right)\left\{-b_{m}z_{m}\left(t\right)+\sum_{n=1}^{l}c_{mn}\left(z_{m}\left(t\right)\right)f_{n}\left(z_{n}\left(t\right)\right)\right.\\ \left.+\sum_{n=1}^{l}d_{mn}\left(z_{m}\left(t\right)\right)f_{n}\left(z_{n}\left(t-\tau_{mn}\left(t\right)\right)\right)+I_{m}\right\}+W_{m}\left(t\right),\;m=1,2,\ldots ,l, \end{array}$$

where $W_{m}\left(t\right)$ is the controller.

Furthermore, similar to the analysis of (5)–(7), we can obtain from (8) that

(9) $$\begin{array}{c} {\dot{q}}_{m}\left(t\right)=-b_{m}h_{m}^{-1}\left(q_{m}\left(t\right)\right)+\sum_{n=1}^{l}c_{mn}^{**}\left(t\right)f_{n}\left(h_{n}^{-1}\left(q_{n}\left(t\right)\right)\right)\\ +\sum_{n=1}^{l}d_{mn}^{**}\left(t\right)f_{n}\left(h_{n}^{-1}\left(q_{n}\left(t-\tau_{mn}\left(t\right)\right)\right)\right)+I_{m}\\ +\frac{W_{m}\left(t\right)}{a_{m}\left(h_{m}^{-1}\left(q_{m}\left(t\right)\right)\right)},\;m=1,2,\ldots ,l, \end{array}$$

where $q_{m}\left(t\right)=h_{m}\left(z_{m}\left(t\right)\right)$, $c_{mn}^{**}\left(t\right)\in co\left[{\tilde{c}}_{mn},{\bar{c}}_{mn}\right]$, $d_{mn}^{**}\left(t\right)\in co\left[{\tilde{d}}_{mn},{\bar{d}}_{mn}\right]$.

Consider the initial conditions of systems (1) and (8) as $r_{m}\left(s\right)=\Upsilon_{m}\left(s\right)$ and $z_{m}\left(s\right)=\Theta_{m}\left(s\right)$, respectively, where −τ≤s≤0. Then, the initial conditions of systems (7) and (9) are $p_{m}\left(s\right)=h_{m}\left(\Upsilon_{m}\left(s\right)\right)$ and $q_{m}\left(s\right)=h_{m}\left(\Theta_{m}\left(s\right)\right)$, respectively, where −τ≤s≤0.

Set errors $E_{m}\left(t\right)=z_{m}\left(t\right)-r_{m}\left(t\right)$, $e_{m}\left(t\right)=q_{m}\left(t\right)-p_{m}\left(t\right)$. It can be obtained from (7) and (9) that

(10) $$\begin{array}{c} {\dot{e}}_{m}\left(t\right)=-b_{m}\left[h_{m}^{-1}\left(q_{m}\left(t\right)\right)-h_{m}^{-1}\left(p_{m}\left(t\right)\right)\right]+\sum_{n=1}^{l}c_{mn}^{**}\left(t\right)f_{n}\left(h_{n}^{-1}\left(q_{n}\left(t\right)\right)\right)\\ -\sum_{n=1}^{l}c_{mn}^{*}\left(t\right)f_{n}\left(h_{n}^{-1}\left(p_{n}\left(t\right)\right)\right)+\sum_{n=1}^{l}d_{mn}^{**}\left(t\right)f_{n}\left(h_{n}^{-1}\left(q_{n}\left(t-\tau_{mn}\left(t\right)\right)\right)\right)\\ -\sum_{n=1}^{l}d_{mn}^{*}\left(t\right)f_{n}\left(h_{n}^{-1}\left(p_{n}\left(t-\tau_{mn}\left(t\right)\right)\right)\right)+\frac{W_{m}\left(t\right)}{a_{m}\left(h_{m}^{-1}\left(q_{m}\left(t\right)\right)\right)}\\ =-b_{m}\left[h_{m}^{-1}\left(q_{m}\left(t\right)\right)-h_{m}^{-1}\left(p_{m}\left(t\right)\right)\right]+\sum_{n=1}^{l}c_{mn}^{**}\left(t\right)g_{n}\left(h_{n}^{-1}\left(e_{n}\left(t\right)\right)\right)\\ +\sum_{n=1}^{l}\left[c_{mn}^{**}\left(t\right)-c_{mn}^{*}\left(t\right)\right]f_{n}\left(h_{n}^{-1}\left(p_{n}\left(t\right)\right)\right)+\sum_{n=1}^{l}d_{mn}^{**}\left(t\right)g_{n}\left(h_{n}^{-1}\left(e_{n}\left(t-\tau_{mn}\left(t\right)\right)\right)\right)\\ +\sum_{n=1}^{l}\left[d_{mn}^{**}\left(t\right)-d_{mn}^{*}\left(t\right)\right]f_{n}\left(h_{n}^{-1}\left(p_{n}\left(t-\tau_{mn}\left(t\right)\right)\right)\right)+v_{m}\left(t\right) \end{array}$$

where $g_{n}\left(h_{n}^{-1}\left(e_{n}\left(t\right)\right)\right)=f_{n}\left(h_{n}^{-1}\left(q_{n}\left(t\right)\right)\right)-f_{n}\left(h_{n}^{-1}\left(p_{n}\left(t\right)\right)\right)$, $g_{n}\left(h_{n}^{-1}\left(e_{n}\left(t-\tau_{mn}\left(t\right)\right)\right)\right)=f_{n}\left(h_{n}^{-1}\left(q_{n}\left(t-\tau_{mn}\left(t\right)\right)\right)\right)-f_{n}\left(h_{n}^{-1}\left(p_{n}\left(t-\tau_{mn}\left(t\right)\right)\right)\right)$ and $v_{m}\left(t\right)=\frac{W_{m}\left(t\right)}{a_{m}\left(h_{m}^{-1}\left(q_{m}\left(t\right)\right)\right)}$. Moreover, the vector form of system (10) can be written as

(11) $$\begin{array}{c} \dot{e}\left(t\right)=-B\left(H^{-1}\left(q\left(t\right)\right)-H^{-1}\left(p\left(t\right)\right)\right)+C^{**}\left(t\right)g\left(H^{-1}\left(e\left(t\right)\right)\right)\\ +\left(C^{**}\left(t\right)-C^{*}\left(t\right)\right)f\left(H^{-1}\left(p\left(t\right)\right)\right)\\ +D^{**}\left(t\right)g\left(H^{-1}\left(e\left(t-\tau \left(t\right)\right)\right)\right)\\ +\left(D^{**}\left(t\right)-D^{*}\left(t\right)\right)f\left(H^{-1}\left(p\left(t-\tau \left(t\right)\right)\right)\right)+v\left(t\right) \end{array}$$

where $e\left(t\right)={\left(e_{1}\left(t\right),e_{2}\left(t\right),\ldots ,e_{l}\left(t\right)\right)}^{T}$, $B=\mathrm{diag}\{b_{1},b_{2},\ldots ,b_{l}\}$, $H^{-1}\left(q\left(t\right)\right)={\left(h_{1}^{-1}\left(q_{1}\left(t\right)\right),h_{2}^{-1}\left(q_{2}\left(t\right)\right),\ldots ,h_{l}^{-1}\left(q_{l}\left(t\right)\right)\right)}^{T}$, $H^{-1}\left(p\left(t\right)\right)={\left(h_{1}^{-1}\left(p_{1}\left(t\right)\right),h_{2}^{-1}\left(p_{2}\left(t\right)\right),\ldots ,h_{l}^{-1}\left(p_{l}\left(t\right)\right)\right)}^{T}$,

$g\left(H^{-1}\left(e\left(t\right)\right)\right)={\left(g_{1}\left(h_{1}^{-1}\left(e_{1}\left(t\right)\right)\right),g_{2}\left(h_{2}^{-1}\left(e_{2}\left(t\right)\right)\right),\ldots ,g_{l}\left(h_{l}^{-1}\left(e_{l}\left(t\right)\right)\right)\right)}^{T}$, $f\left(H^{-1}\left(p\left(t\right)\right)\right)={\left(f_{1}\left(h_{1}^{-1}\left(p_{1}\left(t\right)\right)\right),f_{2}\left(h_{2}^{-1}\left(p_{2}\left(t\right)\right)\right),\ldots ,f_{l}\left(h_{l}^{-1}\left(p_{l}\left(t\right)\right)\right)\right)}^{T}$, $C^{**}\left(t\right)={\left[c_{mn}^{**}\left(t\right)\right]}_{l\times l}$, $C^{*}\left(t\right)={\left[c_{mn}^{*}\left(t\right)\right]}_{l\times l}$, $D^{**}\left(t\right)={\left[d_{mn}^{**}\left(t\right)\right]}_{l\times l}$, $D^{*}\left(t\right)={\left[d_{mn}^{*}\left(t\right)\right]}_{l\times l}$, $v\left(t\right)={\left(v_{1}\left(t\right),v_{2}\left(t\right),\ldots ,v_{l}\left(t\right)\right)}^{T}$.

**Figure 1:**
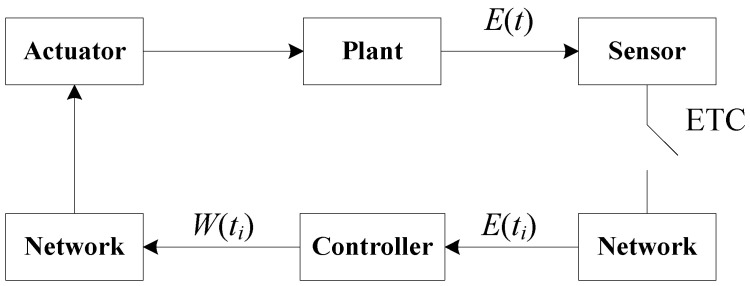
The block diagram of ETC scheme.

Set measured errors between system (1) and system (8) as $ME_{m}\left(t\right)=E_{m}\left(t_{i}\right)-E_{m}\left(t\right)$, $\forall t\in [t_{i},t_{i+1})$, m=1,2,…,l. In the ETC strategy, the state-dependent threshold needs to be set. When the measured errors exceed the threshold, the control will be updated under a new triggering event. It is worth noting that $\lim_{t\to t_{i}^{+}}ME_{m}\left(t\right)=ME_{m}\left(t_{i}\right)=0$, $\lim_{t\to t_{i}^{-}}ME_{m}\left(t\right)=\lim_{t\to t_{i}^{-}}E_{m}\left(t_{i-1}\right)-E_{m}\left(t\right)\neq 0$. Therefore, $ME_{m}\left(t\right)$ are discontinuous at $t=t_{i}$. The schematic of ETC is shown in Figure 1.

Next, the definition of asymptotic synchronization of MCGNNs with time-varying delays is presented as follows.

**Definition** **1.** *If*

(12) $$\lim_{t\to +\infty }{∥z(t)-r(t)∥}_{1}=0,$$

*then MCGNN systems (8) and (1) can achieve asymptotic synchronization, where $z\left(t\right)=(z_{1}\left(t\right)$, $z_{2}\left(t\right),\ldots ,z_{l}{\left(t\right))}^{T}$, $r\left(t\right)=(r_{1}\left(t\right),r_{2}\left(t\right)$, $\ldots ,r_{l}{\left(t\right))}^{T}$.*

## 3. Synchronization of Memristive Cohen-Grossberg Neural Networks

In this section, we will discuss the asymptotic synchronization problem of the MCGNN systems.

We consider the state-feedback controller as follows.

(13) $$W\left(t\right)=-\Lambda E\left(t_{i}\right)-\Gamma \mathrm{sign}\left(E\left(t_{i}\right)\right),t\in \left[t_{i},t_{i+1}\right)$$

where $W\left(t\right)={\left(W_{1}\left(t\right),W_{2}\left(t\right),\ldots ,W_{l}\left(t\right)\right)}^{T}$, $E\left(t_{i}\right)={\left(E_{1}\left(t_{i}\right),E_{2}\left(t_{i}\right),\ldots ,E_{l}\left(t_{i}\right)\right)}^{T}$; $\Lambda =\mathrm{diag}{\left(\Lambda_{1},\Lambda_{2},\ldots ,\Lambda_{l}\right)}^{T}$ is positive definite matrix; $\Gamma =\mathrm{diag}{\left(\Gamma_{1},\Gamma_{2},\ldots ,\Gamma_{l}\right)}^{T}$; $\mathrm{sign}\left(E\left(t_{i}\right)\right)={\left(\mathrm{sign}\left(E_{1}\left(t_{i}\right)\right),\mathrm{sign}\left(E_{2}\left(t_{i}\right)\right),\ldots ,\mathrm{sign}\left(E_{l}\left(t_{i}\right)\right)\right)}^{T}$ represents sign function; and $t_{i}$ is a release instant. Then,

(14) $$\begin{array}{c} v_{m}\left(t\right)=\frac{W_{m}\left(t\right)}{a_{m}\left(h_{m}^{-1}\left(q_{m}\left(t\right)\right)\right)}=-\frac{\Lambda_{m}E_{m}\left(t_{i}\right)}{a_{m}\left(h_{m}^{-1}\left(q_{m}\left(t\right)\right)\right)}-\frac{\Gamma_{m}\mathrm{sign}\left(E_{m}\left(t_{i}\right)\right)}{a_{m}\left(h_{m}^{-1}\left(q_{m}\left(t\right)\right)\right)}. \end{array}$$

Then, the following Theorem 1 and corollaries 1–2 can be obtained on the basis of the state-feedback controller (13).

**Theorem** **1.** *MCGNNs systems (8) and (1) can be synchronized asymptotically under Assumptions 1–3 with the state-feedback controller (13) and the following ETC condition*

(15) $${∥ME(t)∥}_{1}\leq \frac{\eta \min_{1\leq m\leq l}\left(a_{m}^{\left(1\right)}\right)}{\max_{1\leq m\leq l}\left(\Lambda_{m}\right)}\left(\vartheta {∥E(t)∥}_{1}+\mu \right)$$

*for $t\in [t_{i},t_{i+1})$, where*

(16) η∈(0,1]

(17) $$\vartheta =\frac{\min_{1\leq m\leq l}\left(\Lambda_{m}\right)}{\max_{1\leq m\leq l}\left(a_{m}^{\left(2\right)}\right)}-\frac{\lambda }{\min_{1\leq m\leq l}\left(a_{m}^{\left(1\right)}\right)}\geq 0$$

(18) $$\begin{array}{c} \lambda =-\underset{1\leq m\leq l}{min}\left(b_{m}\right)\underset{1\leq m\leq l}{min}\left(a_{m}^{\left(1\right)}\right)+\max_{1\leq m\leq l}\left(L_{m}\right)\max_{1\leq m\leq l}\left(a_{m}^{\left(2\right)}\right){∥\hat{C}∥}_{1}\\ +\frac{\max_{1\leq m\leq l}\left(L_{m}\right)\max_{1\leq m\leq l}\left(a_{m}^{\left(2\right)}\right)}{1-\theta }{∥\hat{D}∥}_{1}>0, \end{array}$$

(19) $$\mu =\sum_{m=1}^{l}\left\{\kappa_{m}-\sum_{n=1}^{l}\left[\left|c_{mn}^{\left(1\right)}-c_{mn}^{\left(2\right)}\right|+\left|d_{mn}^{\left(1\right)}-d_{mn}^{\left(2\right)}\right|\right]M_{n}\right\}$$

(20) $$\left\{\begin{array}{c} \Gamma_{m}>a_{m}^{\left(2\right)}\kappa_{m},\;\mathrm{if}\;\mathrm{sign}\left(E_{m}\left(t\right)\right)\mathrm{sign}\left(E_{m}\left(t_{i}\right)\right)>0,\\ \Gamma_{m}\leq -a_{m}^{\left(2\right)}\kappa_{m},\;\mathrm{otherwise}, \end{array}\right.$$

*and*

(21) $$\kappa_{m}>\sum_{n=1}^{l}\left[\left|c_{mn}^{\left(1\right)}-c_{mn}^{\left(2\right)}\right|+\left|d_{mn}^{\left(1\right)}-d_{mn}^{\left(2\right)}\right|\right]M_{n},$$

*hold.*

**Proof of Theorem 1.**  Consider a Lyapunov functional as

(22) $$V\left(t\right)={∥e(t)∥}_{1}+\sum_{m=1}^{l}\sum_{n=1}^{l}\frac{{\hat{d}}_{mn}}{1-\theta }\int_{t-\tau_{mn}\left(t\right)}^{t}\left|g_{n}\left(h_{n}^{-1}\left(e_{n}\left(s\right)\right)\right)\right|ds$$

For $t\in [t_{i},t_{i+1})$, we can attain the upper right Dini-derivative of V(t) as

(23) $$\begin{array}{c} \dot{V}\left(t\right)\leq {\mathrm{sign}}^{T}\left(e\left(t\right)\right)\dot{e}\left(t\right)\\ +\sum_{m=1}^{l}\sum_{n=1}^{l}{\hat{d}}_{mn}\left[\frac{1}{1-\theta }\left|g_{n}\left(h_{n}^{-1}\left(e_{n}\left(t\right)\right)\right)\right|-\left|g_{n}\left(h_{n}^{-1}\left(e_{n}\left(t-\tau_{mn}\left(t\right)\right)\right)\right)\right|\right]\\ ={\mathrm{sign}}^{T}\left(e\left(t\right)\right)\left\{-B(H^{-1}\left(q\left(t\right)\right)-H^{-1}\left(p\left(t\right)\right))\right.\\ +C^{**}\left(t\right)g\left(H^{-1}\left(e\left(t\right)\right)\right)+\left(C^{**}\left(t\right)-C^{*}\left(t\right)\right)f\left(H^{-1}\left(p\left(t\right)\right)\right)\\ +D^{**}\left(t\right)g\left(H^{-1}\left(e\left(t-\tau \left(t\right)\right)\right)\right)\\ \left.+\left(D^{**}\left(t\right)-D^{*}\left(t\right)\right)f\left(H^{-1}\left(p\left(t-\tau \left(t\right)\right)\right)\right)+v\left(t\right)\right\}\\ +\sum_{m=1}^{l}\sum_{n=1}^{l}{\hat{d}}_{mn}\left[\frac{1}{1-\theta }\left|g_{n}\left(h_{n}^{-1}\left(e_{n}\left(t\right)\right)\right)\right|-\left|g_{n}\left(h_{n}^{-1}\left(e_{n}\left(t-\tau_{mn}\left(t\right)\right)\right)\right)\right|\right]\\ \leq {\mathrm{sign}}^{T}\left(e\left(t\right)\right)\left\{-B(H^{-1}\left(q\left(t\right)\right)-H^{-1}\left(p\left(t\right)\right))\right.\\ +\max_{1\leq m\leq l}\left(L_{m}\right)\max_{1\leq m\leq l}\left(a_{m}^{\left(2\right)}\right){∥\hat{C}∥}_{1}{∥e(t)∥}_{1}+{\mathrm{sign}}^{T}\left(e\left(t\right)\right)\left\{\left(C^{**}\left(t\right)-C^{*}\left(t\right)\right)\right.\\ \times f\left(H^{-1}\left(p\left(t\right)\right)\right)+\left(D^{**}\left(t\right)-D^{*}\left(t\right)\right)f\left(H^{-1}\left(p\left(t-\tau \left(t\right)\right)\right)\right)\\ \left.+v(t)\right\}+\frac{\max_{1\leq m\leq l}\left(L_{m}\right)\max_{1\leq m\leq l}\left(a_{m}^{\left(2\right)}\right)}{1-\theta }{∥\hat{D}∥}_{1}{∥e(t)∥}_{1} \end{array}$$

Since $h_{m}\left(.\right)$ and $h_{m}^{-1}\left(.\right)$ are monotonically increasing, that is to say ${\mathrm{sign}}^{T}\left(e_{m}\left(t\right)\right)(h_{m}^{-1}$$\left(q_{m}\left(t\right)\right)-h_{m}^{-1}\left(p_{m}\left(t\right)\right))=\left|h_{m}^{-1}\left(q_{m}\left(t\right)\right)-h_{m}^{-1}\left(p_{m}\left(t\right)\right)\right|$ and $-{\mathrm{sign}}^{T}\left(e\left(t\right)\right)\Lambda E\left(t\right)=-{\mathrm{sign}}^{T}$(E(t))ΛE(t). Thus, it can be gained that

(24) $$\begin{array}{c} {\mathrm{sign}}^{T}\left(e\left(t\right)\right)\left(-B\left(H^{-1}\left(q\left(t\right)\right)-H^{-1}\left(p\left(t\right)\right)\right)\right)\\ \leq -\underset{1\leq m\leq l}{min}\left(b_{m}\right){∥H^{-1}\left(q\left(t\right)\right)-H^{-1}\left(p\left(t\right)\right)∥}_{1}\\ \leq -\underset{1\leq m\leq l}{min}\left(b_{m}\right)\underset{1\leq m\leq l}{min}\left(a_{m}^{\left(1\right)}\right){∥e(t)∥}_{1} \end{array}$$

and

(25) $$\begin{array}{c} -{\mathrm{sign}}^{T}\left(e\left(t\right)\right)\Lambda E\left(t_{i}\right)=-{\mathrm{sign}}^{T}\left(e\left(t\right)\right)\Lambda \left(E\left(t\right)+ME\left(t\right)\right)\\ =-{\mathrm{sign}}^{T}\left(E\left(t\right)\right)\Lambda E\left(t\right)-{\mathrm{sign}}^{T}\left(e\left(t\right)\right)\Lambda ME\left(t\right)\\ \leq -\min_{1\leq m\leq l}\left(\Lambda_{m}\right){∥E(t)∥}_{1}+\max_{1\leq m\leq l}\left(\Lambda_{m}\right){∥ME(t)∥}_{1}, \end{array}$$

Thus, it can be obtained that

(26) $$\begin{array}{c} {\mathrm{sign}}^{T}\left(e\left(t\right)\right)v\left(t\right)=\sum_{m=1}^{l}\mathrm{sign}\left(e_{m}\left(t\right)\right)\left[-\frac{\Lambda_{m}E_{m}\left(t_{i}\right)}{a_{m}\left(h_{m}^{-1}\left(q_{m}\left(t\right)\right)\right)}-\frac{\Gamma_{m}sign\left(E_{m}\left(t_{i}\right)\right)}{a_{m}\left(h_{m}^{-1}\left(q_{m}\left(t\right)\right)\right)}\right]\\ =-\sum_{m=1}^{l}\mathrm{sign}\left(e_{m}\left(t\right)\right)\frac{\Lambda_{m}\left(E_{m}\left(t\right)+ME_{m}\left(t\right)\right)}{a_{m}\left(h_{m}^{-1}\left(q_{m}\left(t\right)\right)\right)}-\sum_{m=1}^{l}\mathrm{sign}\left(e_{m}\left(t\right)\right)\frac{\Gamma_{m}sign\left(E_{m}\left(t_{i}\right)\right)}{a_{m}\left(h_{m}^{-1}\left(q_{m}\left(t\right)\right)\right)}\\ \leq -\sum_{m=1}^{l}\frac{\Lambda_{m}\left|E_{m}\left(t\right)\right|}{a_{m}\left(h_{m}^{-1}\left(q_{m}\left(t\right)\right)\right)}+\sum_{m=1}^{l}{\frac{\Lambda_{m}\left|ME_{m}\left(t\right)\right|}{a_{m}\left(h_{m}^{-1}\left(q_{m}\left(t\right)\right)\right)}}_{m}-\sum_{m=1}^{l}\mathrm{sign}\left(e_{m}\left(t\right)\right)\frac{\Gamma_{m}sign\left(E_{m}\left(t_{i}\right)\right)}{a_{m}\left(h_{m}^{-1}\left(q_{m}\left(t\right)\right)\right)}\\ \leq -\frac{\min_{1\leq m\leq l}\left(\Lambda_{m}\right)}{\max_{1\leq m\leq l}\left(a_{m}^{\left(2\right)}\right)}{∥E(t)∥}_{1}+\frac{\max_{1\leq m\leq l}\left(\Lambda_{m}\right)}{\min_{1\leq m\leq l}\left(a_{m}^{\left(1\right)}\right)}{∥ME(t)∥}_{1}-\sum_{m=1}^{l}\mathrm{sign}\left(E_{m}\left(t\right)\right)\frac{\Gamma_{m}sign\left(E_{m}\left(t_{i}\right)\right)}{a_{m}\left(h_{m}^{-1}\left(q_{m}\left(t\right)\right)\right)} \end{array}$$

and

(27) $$\begin{array}{c} {\mathrm{sign}}^{T}\left(e\left(t\right)\right)\left\{\left(C^{**}\left(t\right)-C^{*}\left(t\right)\right)f\left(H^{-1}\left(p\left(t\right)\right)\right)\right.\\ \left.+\left(D^{**}\left(t\right)-D^{*}\left(t\right)\right)f\left(H^{-1}\left(p\left(t-\tau \left(t\right)\right)\right)\right)\right\}\\ -\sum_{m=1}^{l}\mathrm{sign}\left(E_{m}\left(t\right)\right)\frac{\Gamma_{m}sign\left(E_{m}\left(t_{i}\right)\right)}{a_{m}\left(h_{m}^{-1}\left(q_{m}\left(t\right)\right)\right)}\\ \leq \sum_{m=1}^{l}\sum_{n=1}^{l}\left[\left|c_{mn}^{\left(1\right)}-c_{mn}^{\left(2\right)}\right|+\left|d_{mn}^{\left(1\right)}-d_{mn}^{\left(2\right)}\right|\right]M_{n}\\ -\sum_{m=1}^{l}\mathrm{sign}\left(E_{m}\left(t\right)\right)\mathrm{sign}\left(E_{m}\left(t_{i}\right)\right)\frac{\Gamma_{m}}{a_{m}\left(h_{m}^{-1}\left(q_{m}\left(t\right)\right)\right)}\\ \leq -\sum_{m=1}^{l}\left\{\kappa_{m}-\sum_{n=1}^{l}\left[\left|c_{mn}^{\left(1\right)}-c_{mn}^{\left(2\right)}\right|+\left|d_{mn}^{\left(1\right)}-d_{mn}^{\left(2\right)}\right|\right]M_{n}\right\}\\ =-\mu <0 \end{array}$$

Then, we can get that

(28) $$\begin{array}{c} \dot{V}\left(t\right)\leq -\underset{1\leq m\leq l}{min}\left(b_{m}\right)\underset{1\leq m\leq l}{min}\left(a_{m}^{\left(1\right)}\right){∥e(t)∥}_{1}\\ +\max_{1\leq m\leq l}\left(L_{m}\right)\max_{1\leq m\leq l}\left(a_{m}^{\left(2\right)}\right){∥\hat{C}∥}_{1}{∥e(t)∥}_{1}\\ -\frac{\min_{1\leq m\leq l}\left(\Lambda_{m}\right)}{\max_{1\leq m\leq l}\left(a_{m}^{\left(2\right)}\right)}{∥E(t)∥}_{1}+\frac{\max_{1\leq m\leq l}\left(\Lambda_{m}\right)}{\min_{1\leq m\leq l}\left(a_{m}^{\left(1\right)}\right)}{∥ME(t)∥}_{1}\\ +\frac{\max_{1\leq m\leq l}\left(L_{m}\right)\max_{1\leq m\leq l}\left(a_{m}^{\left(2\right)}\right)}{1-\theta }{∥\hat{D}∥}_{1}{∥e(t)∥}_{1}-\mu \\ =\frac{\max_{1\leq m\leq l}\left(\Lambda_{m}\right)}{\min_{1\leq m\leq l}\left(a_{m}^{\left(1\right)}\right)}{∥ME(t)∥}_{1}-\frac{\min_{1\leq m\leq l}\left(\Lambda_{m}\right)}{\max_{1\leq m\leq l}\left(a_{m}^{\left(2\right)}\right)}{∥E(t)∥}_{1}+\lambda {∥e(t)∥}_{1}-\mu \\ \leq \frac{\max_{1\leq m\leq l}\left(\Lambda_{m}\right)}{\min_{1\leq m\leq l}\left(a_{m}^{\left(1\right)}\right)}{∥ME(t)∥}_{1}-\left[\frac{\min_{1\leq m\leq l}\left(\Lambda_{m}\right)}{\max_{1\leq m\leq l}\left(a_{m}^{\left(2\right)}\right)}-\frac{\lambda }{\min_{1\leq m\leq l}\left(a_{m}^{\left(1\right)}\right)}\right]{∥E(t)∥}_{1}-\mu \\ \leq \left(\eta -1\right)\left(\vartheta {∥E(t)∥}_{1}+\mu \right)\\ \leq 0 \end{array}$$

It can be obtained that $\lim_{t\to +\infty }{∥e(t)∥}_{1}=0$ according to (22). Then, we have $\frac{1}{\max_{1\leq m\leq l}\left(a_{m}^{\left(2\right)}\right)}\times \lim_{t\to +\infty }{∥z(t)-r(t)∥}_{1}=\frac{1}{\max_{1\leq m\leq l}\left(a_{m}^{\left(2\right)}\right)}\lim_{t\to +\infty }{∥E(t)∥}_{1}\leq \lim_{t\to +\infty }{∥e(t)∥}_{1}=0$, that is to say

$\lim_{t\to +\infty }{∥z(t)-r(t)∥}_{1}=\lim_{t\to +\infty }{∥E(t)∥}_{1}=0$, where $z\left(t\right)={\left(z_{1}\left(t\right),z_{2}\left(t\right),\ldots ,z_{l}\left(t\right)\right)}^{T}$, $r\left(t\right)={\left(r_{1}\left(t\right),r_{2}\left(t\right),\ldots ,r_{l}\left(t\right)\right)}^{T}$.

Thus, the system (8) and system (1) can achieve asymptotic synchronization with the state-feedback controller (13) under the ETC condition (15) on the basis of Definition 1. The proof is finished. □

**Remark** **1.** *At present, the ETC scheme for synchronization of MNNs continues to be widely studied [33,34,35,36,37,38,39,40,41] owing to low computing costs and communication resources of ETC. Accordingly, synchronization of some types of MNNs are achieved, such as quasi-synchronization of delayed MNNs [37], synchronization of delayed MNNs [38], and synchronization of inertial MNNs [39]. However, these ETC schemes were considered in the traditional MNN system [37,38,39], not the MCGNN system. In fact, the amplification function of MCGNNs will increase the difficulty of control and analysis. In this paper, asymptotic synchronization of MCGNNs under the ETC scheme is studied, and it is subsequently demonstrated that ETC can effectively reduce computing costs.*

**Corollary** **1.** *MCGNNs systems (8) and (1) can be synchronized asymptotically under Assumptions 1–3 with the state feedback controller (13) and the following ETC condition.*

(29) $${∥ME(t)∥}_{1}\leq \frac{\min_{1\leq m\leq l}\left(a_{m}^{\left(1\right)}\right)}{\max_{1\leq m\leq l}\left(\Lambda_{m}\right)}\left(\eta \vartheta {∥E(t)∥}_{1}+\mu \right)$$

*for $t\in [t_{i},t_{i+1})$, if *Γ* satisfies (20) and (21), and η, ϑ, λ and μ are same as Theorem 1.*

**Proof of Corollary 1.**  Consider a Lyapunov functional shown in (22). Furthermore, for $t\in [t_{i},t_{i+1})$, we can get the upper right Dini-derivative of V(t) as

(30) $$\begin{array}{c} \dot{V}\left(t\right)\leq -\underset{1\leq m\leq l}{min}\left(b_{m}\right)\underset{1\leq m\leq l}{min}\left(a_{m}^{\left(1\right)}\right){∥e(t)∥}_{1}\\ +\max_{1\leq m\leq l}\left(L_{m}^{\left(1\right)}\right)\max_{1\leq m\leq l}\left(a_{m}^{\left(2\right)}\right){∥\hat{C}∥}_{1}{∥e(t)∥}_{1}\\ -\frac{\min_{1\leq m\leq l}\left(\Lambda_{m}\right)}{\max_{1\leq m\leq l}\left(a_{m}^{\left(2\right)}\right)}{∥E(t)∥}_{1}+\frac{\max_{1\leq m\leq l}\left(\Lambda_{m}\right)}{\min_{1\leq m\leq l}\left(a_{m}^{\left(1\right)}\right)}{∥ME(t)∥}_{1}\\ +\frac{\max_{1\leq m\leq l}\left(L_{m}^{\left(1\right)}\right)\max_{1\leq m\leq l}\left(a_{m}^{\left(2\right)}\right)}{1-\theta }{∥\hat{D}∥}_{1}{∥e(t)∥}_{1}-\mu \\ =\frac{\max_{1\leq m\leq l}\left(\Lambda_{m}\right)}{\min_{1\leq m\leq l}\left(a_{m}^{\left(1\right)}\right)}{∥ME(t)∥}_{1}-\frac{\min_{1\leq m\leq l}\left(\Lambda_{m}\right)}{\max_{1\leq m\leq l}\left(a_{m}^{\left(2\right)}\right)}{∥E(t)∥}_{1}+\lambda {∥e(t)∥}_{1}-\mu \\ \leq \frac{\max_{1\leq m\leq l}\left(\Lambda_{m}\right)}{\min_{1\leq m\leq l}\left(a_{m}^{\left(1\right)}\right)}{∥ME(t)∥}_{1}-\left[\frac{\min_{1\leq m\leq l}\left(\Lambda_{m}\right)}{\max_{1\leq m\leq l}\left(a_{m}^{\left(2\right)}\right)}-\frac{\lambda }{\min_{1\leq m\leq l}\left(a_{m}^{\left(1\right)}\right)}\right]{∥E(t)∥}_{1}-\mu \\ \leq \left(\eta -1\right)\vartheta {∥E(t)∥}_{1}\\ \leq 0 \end{array}$$

□

**Corollary** **2.** *MCGNNs systems (8) and (1) can be synchronized asymptotically under Assumptions 1–3 with the state-feedback controller (13) and the following ETC condition*

(31) $${∥ME(t)∥}_{1}\leq \frac{\min_{1\leq m\leq l}\left(a_{m}^{\left(1\right)}\right)}{\max_{1\leq m\leq l}\left(\Lambda_{m}\right)}\left(\vartheta {∥E(t)∥}_{1}+\eta \mu \right)$$

*for $t\in [t_{i},t_{i+1})$, if *Γ* satisfies (20) and (21), and η, ϑ, λ and μ are same as Theorem 1.*

**Proof of Corollary 2.**  Consider a Lyapunov functional shown in (22). Furthermore, for $t\in [t_{i},t_{i+1})$, we can obtain the upper right Dini-derivative of V(t) as

(32) $$\begin{array}{c} \dot{V}\left(t\right)\leq -\underset{1\leq m\leq l}{min}\left(b_{m}\right)\underset{1\leq m\leq l}{min}\left(a_{m}^{\left(1\right)}\right){∥e(t)∥}_{1}\\ +\max_{1\leq m\leq l}\left(L_{m}^{\left(1\right)}\right)\max_{1\leq m\leq l}\left(a_{m}^{\left(2\right)}\right){∥\hat{C}∥}_{1}{∥e(t)∥}_{1}\\ -\frac{\min_{1\leq m\leq l}\left(\Lambda_{m}\right)}{\max_{1\leq m\leq l}\left(a_{m}^{\left(2\right)}\right)}{∥E(t)∥}_{1}+\frac{\max_{1\leq m\leq l}\left(\Lambda_{m}\right)}{\min_{1\leq m\leq l}\left(a_{m}^{\left(1\right)}\right)}{∥ME(t)∥}_{1}\\ +\frac{\max_{1\leq m\leq l}\left(L_{m}^{\left(1\right)}\right)\max_{1\leq m\leq l}\left(a_{m}^{\left(2\right)}\right)}{1-\theta }{∥\hat{D}∥}_{1}{∥e(t)∥}_{1}-\mu \\ =\frac{\max_{1\leq m\leq l}\left(\Lambda_{m}\right)}{\min_{1\leq m\leq l}\left(a_{m}^{\left(1\right)}\right)}{∥ME(t)∥}_{1}-\frac{\min_{1\leq m\leq l}\left(\Lambda_{m}\right)}{\max_{1\leq m\leq l}\left(a_{m}^{\left(2\right)}\right)}{∥E(t)∥}_{1}+\lambda {∥e(t)∥}_{1}-\mu \\ \leq \frac{\max_{1\leq m\leq l}\left(\Lambda_{m}\right)}{\min_{1\leq m\leq l}\left(a_{m}^{\left(1\right)}\right)}{∥ME(t)∥}_{1}-\left[\frac{\min_{1\leq m\leq l}\left(\Lambda_{m}\right)}{\max_{1\leq m\leq l}\left(a_{m}^{\left(2\right)}\right)}-\frac{\lambda }{\min_{1\leq m\leq l}\left(a_{m}^{\left(1\right)}\right)}\right]{∥E(t)∥}_{1}-\mu \\ \leq (\eta -1)\mu \\ \leq 0 \end{array}$$

□

**Remark** **2.** *In recent years, many papers on synchronization of MCGNNs have emerged [24,25,26,27,28]. It can be observed that achieving the synchronization of MCGNNs is extremely important due to the meaning synchronization of multiple classes of neural networks. However, to date there are no studies that employ the ETC scheme to achieve synchronization of MCGNNs, as far as we know. Indeed, the amplification function of MCGNNs will increase the difficulty of control and analysis. In this paper, asymptotic synchronization of MCGNNs is realized via ETC, and ETC is demonstrated to effectively reduce computing costs through decreased update times of the controller.*

## 4. Numerical Simulations

In this section, we provide an example to verify the validity of the obtained results.

Consider a drive MCGNN system as

(33) $$\left\{\begin{array}{c} \begin{array}{c} {\dot{r}}_{1}\left(t\right)=\left(1+0.02\sin(r_{1}\left(t\right))\right)\left\{-8.6r_{1}\left(t\right)+\sum_{n=1}^{2}c_{1n}\left(r_{1}\left(t\right)\right)f_{n}\left(r_{n}\left(t\right)\right))\right.\\ \left.+\sum_{n=1}^{2}d_{1n}\left(r_{1}\left(t\right)\right)f_{n}\left(r_{n}\left(t-\tau_{1n}\left(t\right)\right)\right)\right\},\; \end{array}\\ \begin{array}{c} {\dot{r}}_{2}\left(t\right)=\left(1+0.01\cos(r_{2}\left(t\right))\right)\left\{-9r_{2}\left(t\right)+\sum_{n=1}^{2}c_{2n}\left(r_{2}\left(t\right)\right)f_{n}\left(r_{n}\left(t\right)\right))\right.\\ \left.+\sum_{n=1}^{2}d_{2n}\left(r_{2}\left(t\right)\right)f_{n}\left(r_{n}\left(t-\tau_{2n}\left(t\right)\right)\right)\right\},\; \end{array} \end{array}\right.$$

where $\tau_{mn}\left(t\right)=\left(e^{t}-1\right)/\left(e^{t}+1\right)$, $f_{n}\left(w\right)=\left(|w+1|-|w-1|\right)/2$, m,n=1,2; memristive connection weights:

$c_{11}\left(r_{1}\left(t\right)\right)=\left\{\begin{array}{cc} 0.18, & |r_{1}\left(t\right)|\leq 2.5,\\ 0.45, & |r_{1}\left(t\right)|>2.5, \end{array}\right.$ $c_{12}\left(r_{1}\left(t\right)\right)=\left\{\begin{array}{cc} -0.23, & |r_{1}\left(t\right)|\leq 2.5,\\ -0.96, & |r_{1}\left(t\right)|>2.5, \end{array}\right.$

$c_{21}\left(r_{2}\left(t\right)\right)=\left\{\begin{array}{cc} 0.09, & |r_{2}\left(t\right)|\leq 2.5,\\ 0.26, & |r_{2}\left(t\right)|>2.5, \end{array}\right.$ $c_{22}\left(r_{2}\left(t\right)\right)=\left\{\begin{array}{cc} 1.06, & |r_{2}\left(t\right)|\leq 2.5,\\ 0.76, & |r_{2}\left(t\right)|>2.5, \end{array}\right.$

$d_{11}\left(r_{1}\left(t\right)\right)=\left\{\begin{array}{cc} 0.85, & |r_{1}\left(t\right)|\leq 2.5,\\ 1.61, & |r_{1}\left(t\right)|>2.5, \end{array}\right.$ $d_{12}\left(r_{1}\left(t\right)\right)=\left\{\begin{array}{cc} 1.52, & |r_{1}\left(t\right)|\leq 2.5,\\ 0.34, & |r_{1}\left(t\right)|>2.5, \end{array}\right.$

$d_{21}\left(r_{2}\left(t\right)\right)=\left\{\begin{array}{cc} 0.86, & |r_{2}\left(t\right)|\leq 2.5,\\ 1.63, & |r_{2}\left(t\right)|>2.5, \end{array}\right.$ $d_{22}\left(r_{2}\left(t\right)\right)=\left\{\begin{array}{cc} -1.03, & |r_{2}\left(t\right)|\leq 2.5,\\ -0.59, & |r_{2}\left(t\right)|>2.5, \end{array}\right.$

Furthermore, the response system can be described as

(34) $$\left\{\begin{array}{c} \begin{array}{c} {\dot{z}}_{1}\left(t\right)=\left(1+0.02\sin(z_{1}\left(t\right))\right)\left\{-8.6z_{1}\left(t\right)+\sum_{n=1}^{2}c_{1n}\left(z_{1}\left(t\right)\right)f_{n}\left(z_{n}\left(t\right)\right))\right.\\ \left.+\sum_{n=1}^{2}d_{1n}\left(z_{1}\left(t\right)\right)f_{n}\left(z_{n}\left(t-\tau_{1n}\left(t\right)\right)\right)\right\}-\Lambda_{1}E_{1}\left(t_{i}\right)-\Gamma_{1}sign\left(E_{1}\left(t_{i}\right)\right),t\in \left[t_{i},t_{i+1}\right)\; \end{array}\\ \begin{array}{c} {\dot{z}}_{2}\left(t\right)=\left(1+0.01\cos(z_{2}\left(t\right))\right)\left\{-9z_{2}\left(t\right)+\sum_{n=1}^{2}c_{2n}\left(z_{2}\left(t\right)\right)f_{n}\left(z_{n}\left(t\right)\right))\right.\\ \left.+\sum_{n=1}^{2}d_{2n}\left(z_{2}\left(t\right)\right)f_{n}\left(z_{n}\left(t-\tau_{2n}\left(t\right)\right)\right)\right\}-\Lambda_{2}E_{2}\left(t_{i}\right)-\Gamma_{2}sign\left(E_{2}\left(t_{i}\right)\right),t\in \left[t_{i},t_{i+1}\right)\; \end{array} \end{array}\right.$$

Then $0.98\leq a_{1}\left(x\right)\leq 1.02$, $0.99\leq a_{1}\left(x\right)\leq 1.01$ for ∀x∈ℜ.${\dot{\tau }}_{mn}\left(t\right)\leq 0.5$; $\left|f_{n}\left(s_{1}\right)\right|\leq 1$ and $\left|f_{n}\left(s_{2}\right)-f_{n}\left(s_{3}\right)\right|\leq \left|s_{2}-s_{3}\right|$ for $\forall s_{1},s_{2},s_{3}\in ℜ$. Thus, we can set $a_{1}^{\left(1\right)}=0.98$, $a_{1}^{\left(2\right)}=1.02$, $a_{2}^{\left(1\right)}=0.99$, $a_{2}^{\left(2\right)}=1.01$, θ=0.5; $M_{n}=1$; $L_{n}=1$. Moreover, we can get $\hat{C}=\left[\begin{array}{cc} 0.45 & 0.96\\ 0.26 & 1.06 \end{array}\right]$ $\hat{D}=\left[\begin{array}{cc} 1.61 & 1.52\\ 1.63 & 1.03 \end{array}\right]$ and ${∥\hat{C}∥}_{1}=2.02$, ${∥\hat{D}∥}_{1}=3.24$. Further, we can gain λ=0.242. Moreover, we choose $\Lambda =\mathrm{diag}{\left(0.90,0.95\right)}^{T}$, such that ϑ=0.635≥0.

Combining with

(35) $$\sum_{n=1}^{2}\left[\left|c_{1n}^{\left(1\right)}-c_{1n}^{\left(2\right)}\right|+\left|d_{1n}^{\left(1\right)}-d_{1n}^{\left(2\right)}\right|\right]M_{n}=2.94$$

and

(36) $$\sum_{n=1}^{2}\left[\left|c_{1n}^{\left(1\right)}-c_{1n}^{\left(2\right)}\right|+\left|d_{1n}^{\left(1\right)}-d_{1n}^{\left(2\right)}\right|\right]M_{n}=2.94.$$

We can choose $\kappa_{1}=2.95$, $\kappa_{2}=1.70$, μ=0.03. Furthermore, the following relations can be obtained.

(37) $$\left\{\begin{array}{c} \Gamma_{1}=3.1>3.009,\;\mathrm{if}\;\mathrm{sign}\left(E_{1}\left(t\right)\right)\mathrm{sign}\left(E_{1}\left(t_{i}\right)\right)>0,\\ \Gamma_{1}=-3.1\leq -3.009,\;\mathrm{otherwise}, \end{array}\right.$$

and

(38) $$\left\{\begin{array}{c} \Gamma_{2}=1.8>1.717,\;\mathrm{if}\;\mathrm{sign}\left(E_{2}\left(t\right)\right)\mathrm{sign}\left(E_{2}\left(t_{i}\right)\right)>0,\\ \Gamma_{2}=-1.8\leq -1.717,\;\mathrm{otherwise}, \end{array}\right.$$

Thus, we can have the following ETC condition

(39) $${∥ME(t)∥}_{1}\leq 1.03\eta \left(0.635{∥E(t)∥}_{1}+0.03\right)$$

for $t\in [t_{i},t_{i+1})$, η∈(0,1].

**Figure 2:**
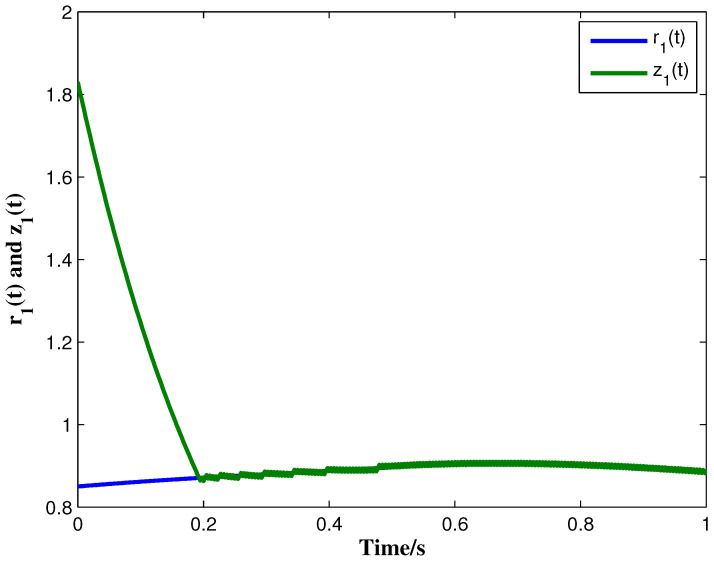
State trajectories of $r_{1}\left(t\right)$ and $z_{1}\left(t\right)$ with controller and ETC.

**Figure 3:**
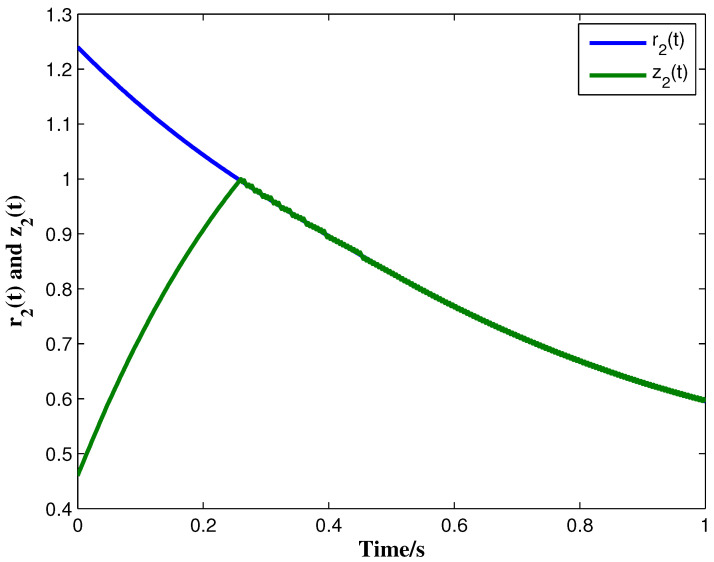
State trajectories of $r_{2}\left(t\right)$ and $z_{2}\left(t\right)$ with controller and ETC.

**Figure 4:**
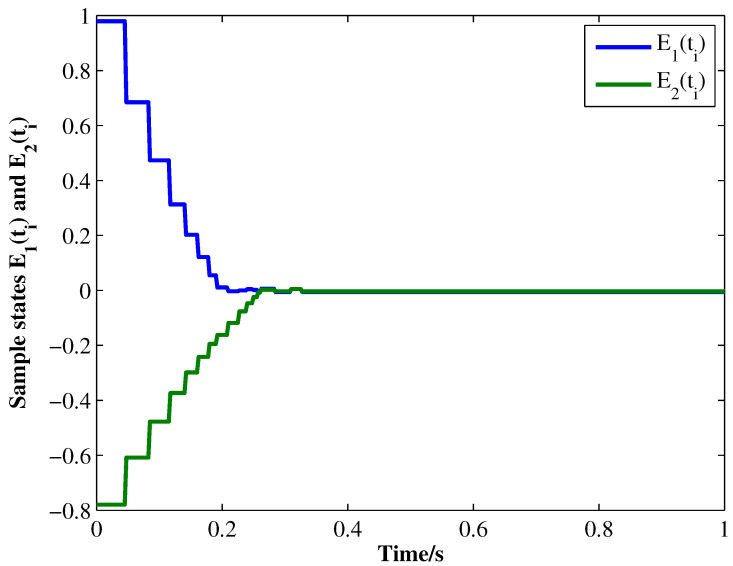
Sample errors $E_{1}\left(t_{i}\right)$ and $E_{2}\left(t_{i}\right)$ between systems (33) and (34) under ETC condition (39) with η=0.5.

**Figure 5:**
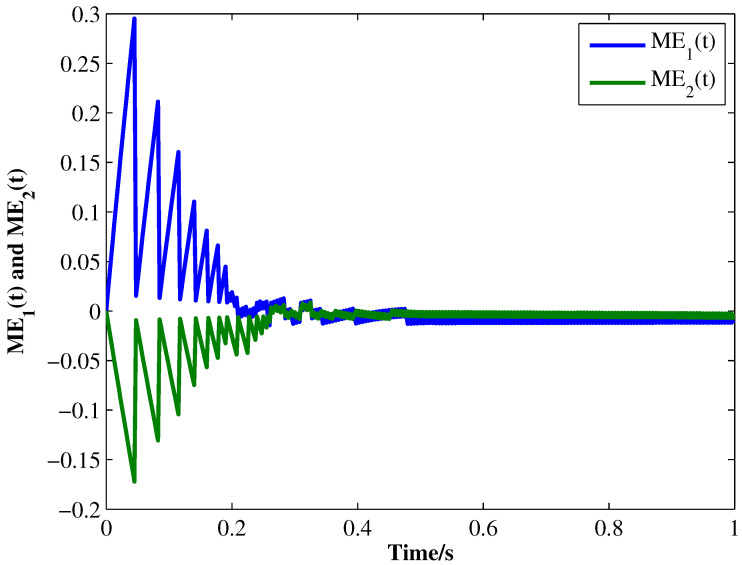
Measured errors $ME_{1}\left(t\right)$ and $ME_{2}\left(t\right)$ between systems (33) and (34) under ETC condition (39) with η=0.5.

**Figure 6:**
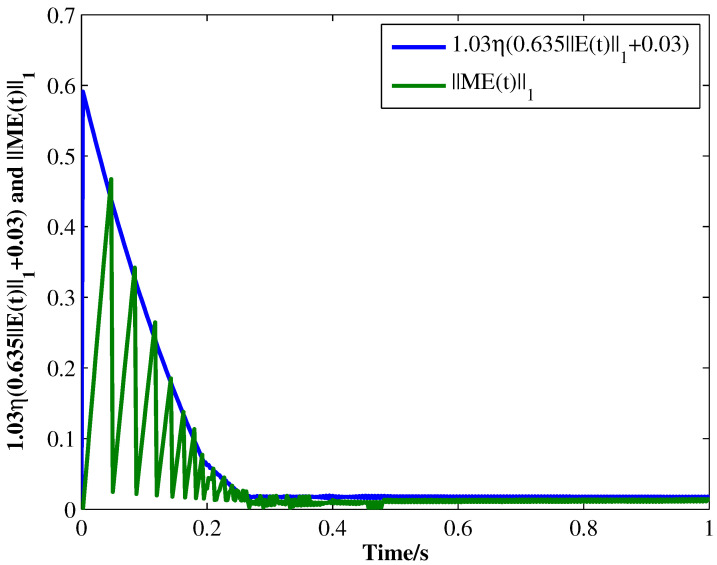
The relationship between 1 and Norm ${\left||ME\left(t\right)|\right|}_{1}$ and the threshold $1.03\eta \left(0.635{∥E(t)∥}_{1}+0.03\right)$ under ETC condition (39) with η=0.5.

**Figure 7:**
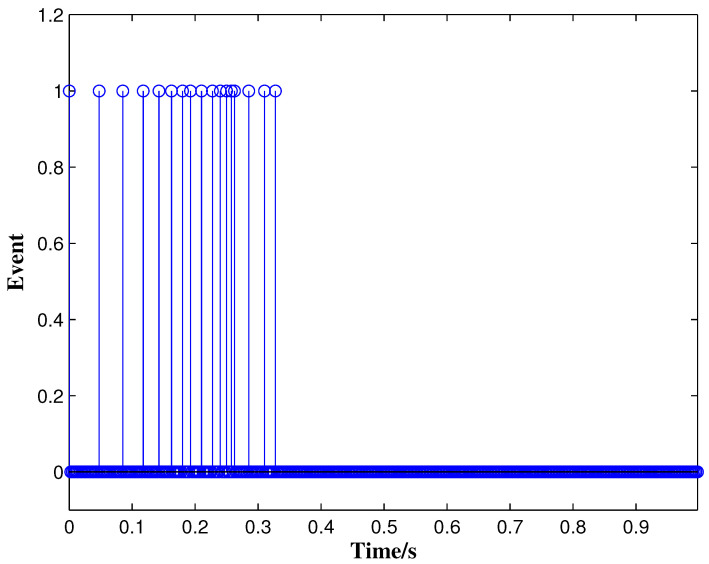
Event-triggered instants under ETC condition (39) with η=0.5.

**Figure 8:**
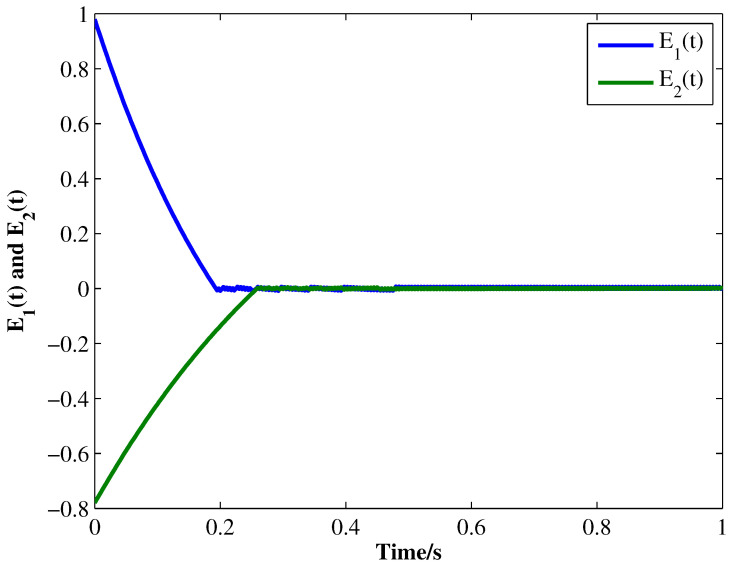
Synchronization errors $E_{1}\left(t\right)$ and $E_{2}\left(t\right)$ between systems (33) and (34) under ETC condition (39) with η=0.5.

**Figure 9:**
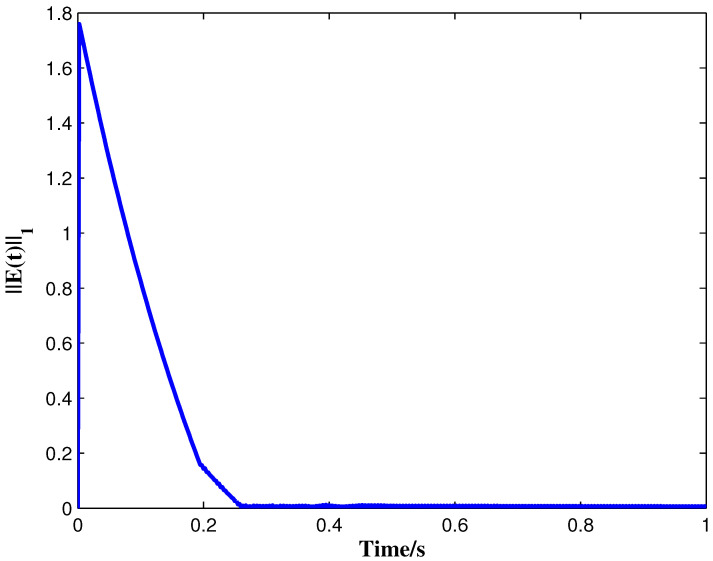
Synchronization errors ${\left||E\left(t\right)|\right|}_{1}$ between systems (33) and (34) under ETC condition (39) with η=0.5.

From the conditions of Theorem 1, we can identify that the drive MCGNN system (33) and the response system (34) can achieve asymptotic synchronization under the ETC condition (39). Consider the initial conditions of systems (33) and (34) as $r\left(s\right)={\left(0.85,1.24\right)}^{T}$ and $z\left(s\right)={\left(1.83,0.46\right)}^{T}$, respectively, η=0.5, then the simulation results are shown in Figure 2, Figure 3, Figure 4, Figure 5, Figure 6, Figure 7, Figure 8 and Figure 9. As shown in Figure 2, Figure 3, Figure 8 and Figure 9, MCGNNs systems (33) and (34) can be synchronized asymptotically under the ETC condition (39). Sample error $E_{m}\left(t_{i}\right)$ and measured error $ME_{m}\left(t\right)$ are shown in Figure 4 and Figure 5, respectively. When the measured error $ME_{m}\left(t\right)$ breaches the ETC condition, that is 1-Norm ${\left||ME\left(t\right)|\right|}_{1}$ exceeds the threshold $1.03\eta \left(0.635{∥E(t)∥}_{1}+0.03\right)$ under the ETC condition (39), the event is triggered, as shown in Figure 6. From Figure 7, it can be found that the ETC scheme can effectively reduce the update times of the controller.

## 5. Conclusions

In this paper, a type of state-feedback controller and several ETC conditions are designed. Under ETC conditions and the state controller, we obtain some sufficient conditions to achieve the asymptotic synchronization of MCGNNs. The results show that MCGNNs under the ETC scheme can effectively reduce the update times of controllers and computing costs.

Although there exist many papers on synchronization of MCGNNs [24,25,26,27,28] and network control schemes including ETC [43,44,45,46,47,48], there is no work yet that has employed the ETC scheme to achieve synchronization of MCGNNs, as far as we know. In this paper, asymptotic synchronization of MCGNNs is realized via ETC for the first time. Therefore, the obtained result can extend upon the existing results [24,25,26,27,28,43,44,45,46,47,48]. In future research, other types of MNN synchronization [49,50] via the ETC scheme will be considered to investigate.
